# Supplementary material for: The anti‐hypertensive drug prazosin inhibits glioblastoma growth via the PKCδ‐dependent inhibition of the AKT pathway
Source: EMBO Mol Med. 2016 Apr 4;8(5):511–26. doi: 10.15252/emmm.201505421 (PMC5130115; doi:10.15252/emmm.201505421)
Supplement: Supplementary file 1 — Appendix [file EMMM-8-511-s001.pdf]

APPENDIX

Appendix Table S1

List of antibodies used :

- PKCdelta, sc-937 (Santa Cruz Biotechnologies), 1 :500, 20 references listed at http://www.citeab.com/antibodies/823084-sc-937-pkc-c-20/publications
- phospho-p42/p44 ERK ref 9106S (Cell Signaling), 1 :1000, Fonseca, B.D. et al. (2011) J Biol Chem 286, 27111-22.
- p42/p44 ERK 06-182 DAM1472295 (Millipore), 1 :5000, List of 80 publications at: http://www.merckmillipore.com/FR/fr/product/Anti-MAP-Kinase-12-%28Erk12%29-Antibody%2C-CT,MM_NF-06-182?cid=BI-XX-BRC-D-CIAB-ANTI-B032-1308&bd=1#seeallref
- phospho-AKT, ref. 4060 (Cell Signaling), 1 :1000, references listed at: http://1degreebio.org/reagents/product/808715/?qid=1107994
- AKT, ref. 9272 (Cell Signaling), 1 :1000, references listed at: http://1degreebio.org/reagents/product/809569/?qid=1107993
- cleaved caspase-3, ref 9661, (Cell Signaling), 1 :1000, references listed at: http://1degreebio.org/reagents/product/862644/?qid=1107997
- caspase-3, ref 9662, (Cell Signaling), 1 :1000, references listed at: http://www.cellsignal.com/products/primary-antibodies/caspase-3-antibody/9662
- Caspase-9, ref # 9502, (Cell Signaling), 1 :1000, Yu, W. et al. (2003) Cancer Res. 63 (10), 2483-91.
- Cyclin D1, ref 2922 (Cell Signaling), 1 :1000, Adon, A.M. et al. (2010) Mol Cell Biol 30, 694-710.
- Cyclin D3, ref 2936(Cell Signaling), 1 :1000, Bartkova, J. et al. (1998) Oncogene 17, 1027-37.
- CDK2, ref2546, (Cell Signaling), 1 :1000, Zhang, S. et al. (2009) Mol Cancer Res 7, 570-80.
- beta-catenin ref 610154 (BD Biociences), 1 :8000, references listed at: http://1degreebio.org/reagents/product/868930/?qid=1108099
- alpha-actin ref MAB1501R (Millipore), 1 :10000, Over 70 references at: http://www.merckmillipore.com/FR/fr/product/Anti-Actin-Antibody%2C-near-a.a.-50-70%2C-clone-C4,MM_NF-MAB1501R?cid=BI-XX-BRC-A-NANT-ANTI-B033-1308#seeallref
- CD133/1-APC, CD133/2-APC, 0.05µg/106 cells, ref #130-090-854 (Miltenyi Biotech), references listed at: http://1degreebio.org/reagents/product/751427/?qid=1108133
- Ki67, ref #MA1-90584 (Thermo Scientific), 1 :200, references listed at: http://1degreebio.org/reagents/product/424214/publications/?qid=1108139
- EGFR-Alexa 488 ref 352907, (Biolegend), 2µg/106 cells, Li, Y et al, Development. 2013 Oct;140(19):3965-76.
- Alexa 488-conjugated goat anti-rabbit, ref A11008, 1 :5000, Molecular Probes, Dufour C et al, 2009, Stem Cells, 27(10):2373-82.
- CY3-conjugated donkey anti-rabbit, ref 711-166-152, 1 :5000, Jackson Immunoresearch, Dufour C et al, 2009, Stem Cells, 27(10):2373-82.
- CY3-conjugated goat anti-mouse, ref PA43002, 1 :500, GE Healthcare, Dufour C et al, 2009, Stem Cells, 27(10):2373-82.
- anti-mouse IgG-HRP, ref NA 931, 1 :10000, GE Healthcare, Thirant C et al, 2012, Stem Cells, 30(5):845-53.
- anti-rabbit IgG, ref NA 9340, 1 :10000GE Healthcare.
- ß3-Tubulin, ref #MAB1637, 1 :1000, Millipore, references listed at: http://1degreebio.org/reagents/product/851166/publications/?qid=1109615
- GFAP, ref 173 011, Synaptic Systems, 1 : 500, Ott C, et al. Molecular Medicine, 2015, Sep 1. doi: 10.2119/molmed.2015.00192.
- O4, MAB1326, R&D, 1 µg/ml, Hong S, et al. J. Neurochem., 2007;104(2):316-24.
