# Supplementary material for: The anti‐hypertensive drug prazosin inhibits glioblastoma growth via the PKCδ‐dependent inhibition of the AKT pathway
Source: EMBO Mol Med. 2016 Apr 4;8(5):511–26. doi: 10.15252/emmm.201505421 (PMC5130115; doi:10.15252/emmm.201505421)
Supplement: Supplementary file 2 — Expanded View Figures PDF [file EMMM-8-511-s002.pdf]

Expanded View Figures

Figure EV1. Prazosin induces GIC apoptosis.

A Flow cytometry analysis for CD15, Annexin V and DAPI in prazosin-treated GBM44 cells. Prazosin induces apoptosis in both $CD15^+$ and $CD15^-$ glioblastoma cells.

B TUNEL staining shows increased numbers of tumor cells undergoing apoptosis following *in vivo* prazosin treatment of GBM44-bearing mice. Right panel: quantification of TUNEL-positive glioblastoma cells in vehicle- versus prazosin-treated mice. Protocol design is schematized in Fig 2A. Mice were sacrificed 48 h after the last prazosin injection. Scale bar: 50 μm. Results are presented as mean ± SD in biological quadruplicates from three independent experiments. $^*P < 0.05$, two-sided Mann–Whitney *U*-test.

C *In vivo* prazosin treatment does not alter angiogenesis. Representative H&E images of tumors initiated with GBM44 grafting. Mice were treated according to the protocol depicted in Fig 2A and sacrificed 2 days after the last prazosin injection. Arrowheads point to blood vessels. Scale bar: 50 μm.

D Viability analysis of GICs that escaped prazosin treatment. GICs having escaped a first prazosin treatment are responsive to a second prazosin treatment at 30 μM. GICs were treated with prazosin for 72 h. The medium was then replaced with fresh medium, and the cells were allowed to recover for 2 weeks prior to be exposed to prazosin for 72 h.

© 2016 The Authors

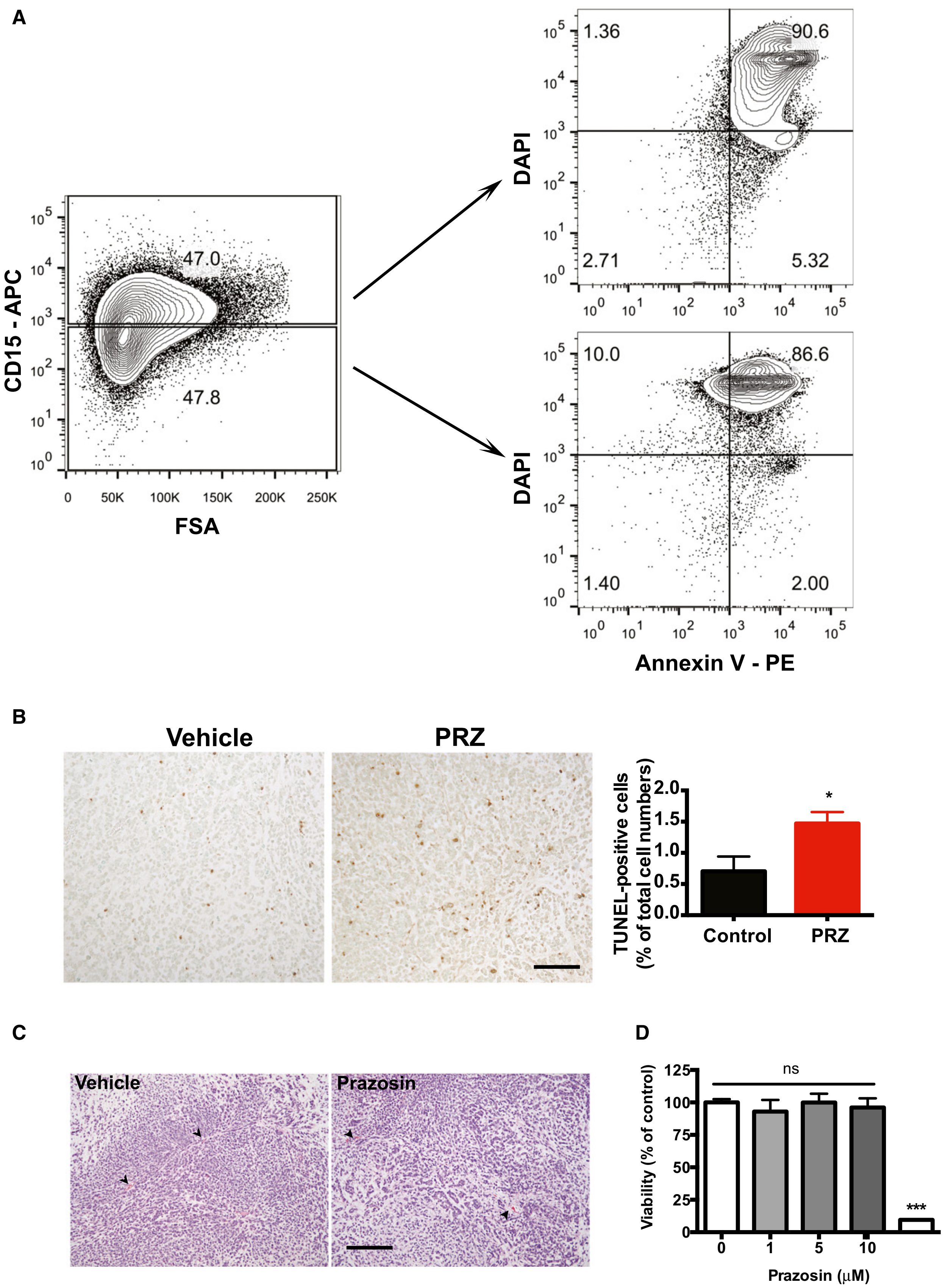

Figure EV1.

© 2016 The Authors

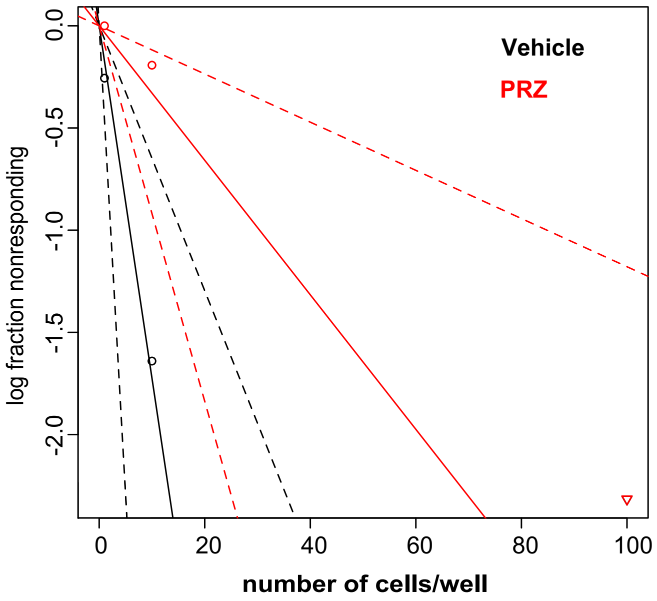

Figure EV2. Extreme limiting dilution assay of GBM44.
Prazosin inhibits the sphere-forming capability of GICs. Extreme limiting dilution assay. GBM44 cells were seeded in presence of vehicle or 10 μM prazosin (PRZ). Sphere formation was scored 21 days post-seeding. Control = 1/6.32 (lower 15.9, upper 2.72); prazosin 1/248 (lower 85.3, upper 11.4), $n = 6$, $P = 0.0331$, overall test for difference in stem cell number between groups.

© 2016 The Authors

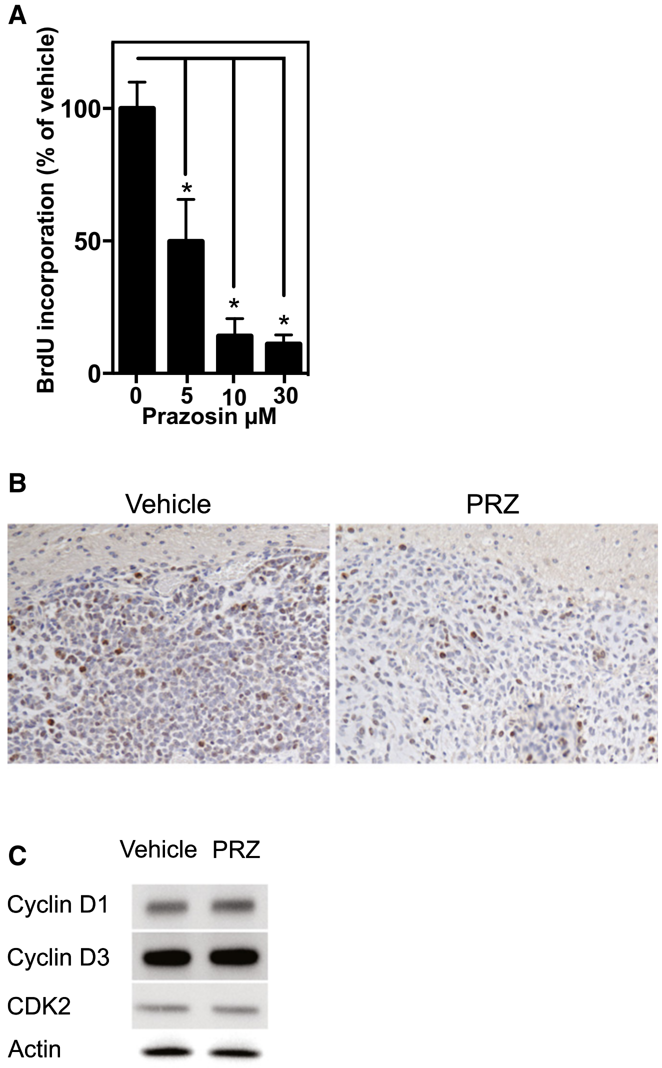

Figure EV3. Prazosin-induced apoptosis is accompanied with reduced GIC proliferation.

Protocol design is schematized in Fig 3A. Mice were sacrificed 48 h after the last prazosin injection.

A BrdU incorporation analysis of GICs treated with prazosin for 24 h. *P = 0.0286, n = 4, two-sided Mann–Whitney U-test.

B Decreased Ki67 immunostaining in tumors following *in vivo* prazosin treatment compared to control. The same results were obtained from 3 independent brain slices taken from more than 6 independent experiments.

C Prazosin does not alter the expression of cyclin D1, cyclin D2, and CDK2 proteins, which are required for G1/S transition. Results are from one Western blot out of three independent experiments giving similar results.

 © 2016 The Authors

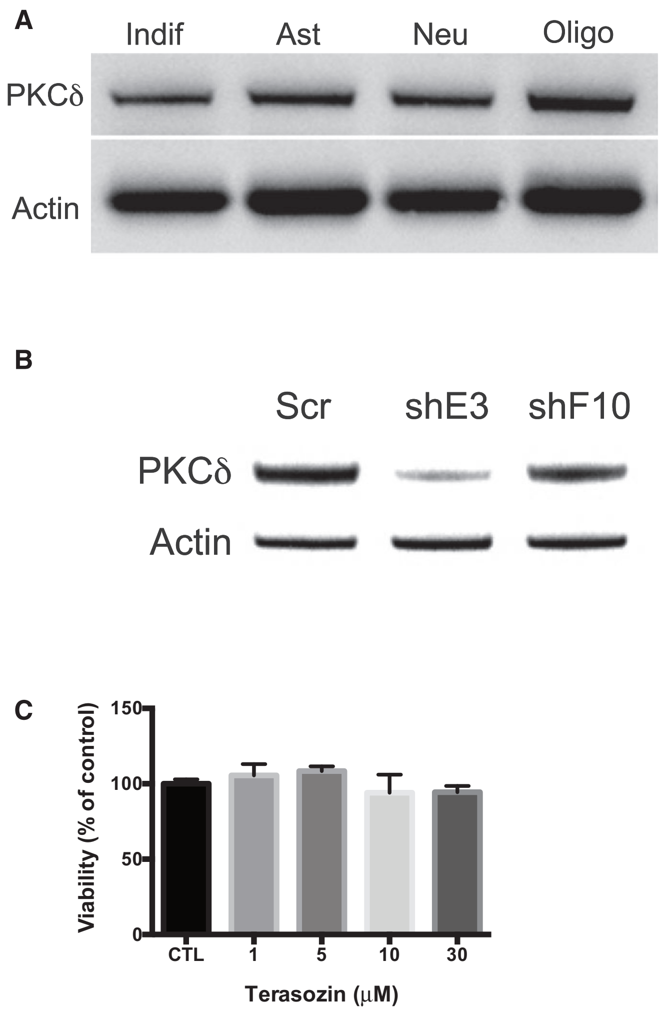

Figure EV4. PKCδ expression and silencing in GIC and differentiated cells.

A Immunoblotting for PKCδ in undifferentiated and differentiated GICs along the astroglial, neuronal and oligodendroglial lineages.

B Immunoblotting for PKCδ in GICs transduced with shRNA for PKCδ (shE3 and shF10) as compared to GICs transduced shScrambled (Scr). shE3 was used in all experiments presented.

C Viability analysis of GICs used to assay the effects of terazosin on AKT phosphorylation (reported on Fig 5H, right panel). Blots are representative of one sample out of three biological replicates analyzed in three independent experiments.

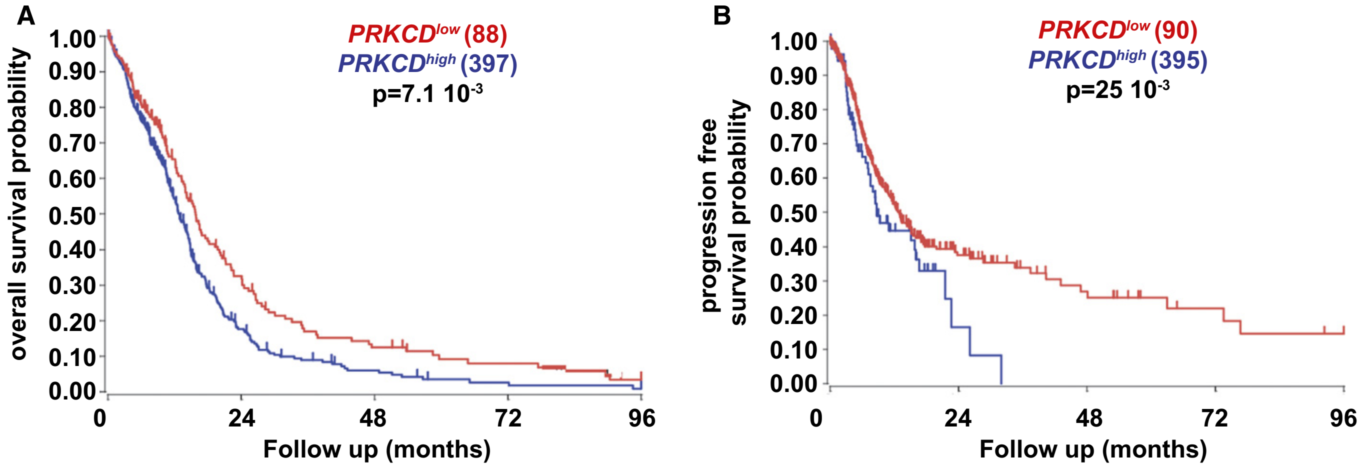

Figure EV5. PKCδ expression is associated with a poorer prognosis in human patients.

A, B Analysis of the TCGA dataset revealed that *PRKCD* transcript levels are inversely correlated with the overall (A) and progression-free (B) survival of adult glioblastoma patients (the analysis was restricted to the samples of untreated patients, log-rank Mantel–Cox test, TCGA cohort).

© 2016 The Authors
