# Supplementary figures and images for: The anti‐hypertensive drug prazosin inhibits glioblastoma growth via the PKCδ‐dependent inhibition of the AKT pathway
Source: EMBO Mol Med. 2016 Apr 4;8(5):511–26. doi: 10.15252/emmm.201505421 (PMC5130115; doi:10.15252/emmm.201505421)

Caspase 3 blots related to Fig 4A

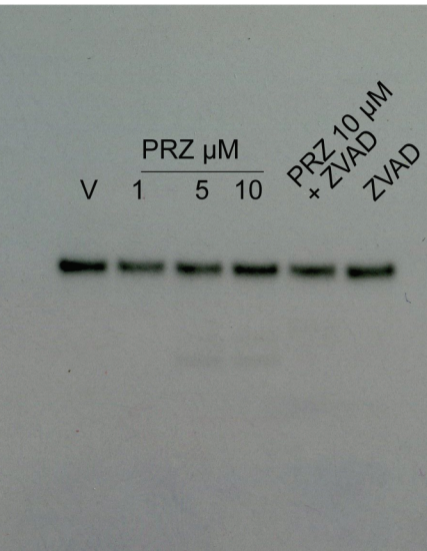

ERK and P-ERK blots related to Fig 4H

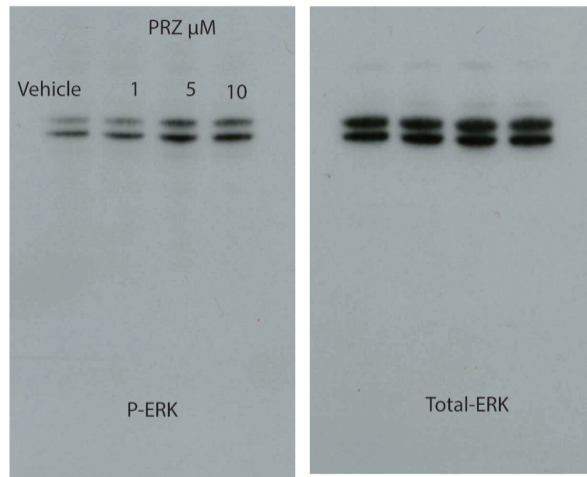

Supplement: Supplementary file 4 — Source Data for Figure 4 [file EMMM-8-511-s003.pdf]

beta-catenin blots related to Fig 5J

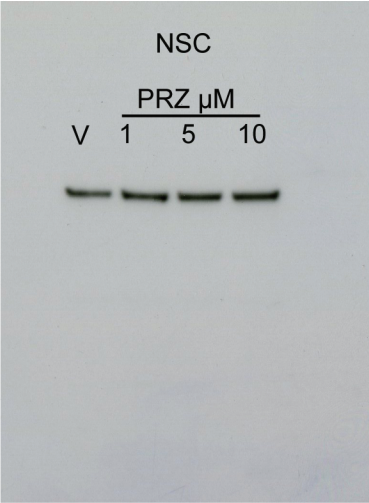

AKT blots related to Fig 5L

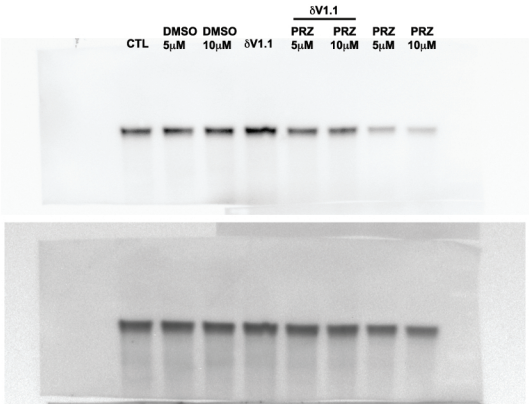

Supplement: Supplementary file 5 — Source Data for Figure 5 [file EMMM-8-511-s004.pdf]
